# Comparative analysis of fit, mechanical properties, and surface characteristics in subtractive- and additive-manufactured zirconia crowns

**DOI:** 10.1186/s12903-025-06561-7

**Published:** 2025-08-01

**Authors:** Su-Min Cho, Ji-Man Park, Alexis Ioannidis, Ryan Jin Young Kim, Hye-Min Chung

**Affiliations:** 1 Dental Research Institute, Seoul National University School of Dentistry, Seoul, Republic of Korea; 2 Department of Prosthodontics & Dental Research Institute, Seoul National University School of Dentistry, Seoul, Republic of Korea; 3 Clinic of Fixed and Removable Prosthodontics and Dental Material Science, University of Zurich, Zurich, Switzerland

**Keywords:** Zirconia, 3D-printed crown, Dental prosthesis fit, Physical properties, Mechanical properties

## Abstract

**Background:**

This study presents different zirconia additive manufacturing (AM) materials and technologies while assessing the fit, hardness, and shear bond strength of crowns produced by AM and subtractive manufacturing (SM) methods, as well as their surface characteristics.

**Methods:**

Zirconia crowns were fabricated using a 5-axis SM and five AM approaches, including four digital light processing principles and one stereolithography (SLA) technique. Each method utilized varying slurry delivery and light-curing mechanisms. The replica technique measured marginal and internal gaps (axial, line angle, and occlusal) between the crowns and abutments. The Vickers hardness and shear bond strength of crowns bonded with resin cement were assessed. Surface characteristics were analyzed with scanning electron microscopy (SEM) post-printing and sandblasting. The fit, hardness, and shear bond strength of crowns were manufactured through AM and SM methods. Sixty crowns were fabricated (10 per group). Statistical analysis was conducted using one-way analysis of variance (ANOVA) with Tukey post-hoc testing (α = 0.05).

**Results:**

The marginal fits were 48.45 µm and 42.83 to 81.95 µm for the S and AM groups, respectively. Significant differences were observed between groups (< 0.001), although all measurements fell within the clinical acceptance range (120 µm). The Vickers hardness measurements revealed that the SM group had a hardness of 1473.87 HV, whereas those of the AM groups ranged from 1441.94 to 1532.53 HV, showing statistically significant differences (*P* < 0.001). Shear bond strength measurements displayed 7.97 MPa and 6.97 to 8.97 MPa for the SM and AM groups, respectively, with no significant differences between the groups. SEM analysis of the crown surfaces revealed agglomerated zirconia particles with various grooves after sandblasting.

**Conclusions:**

Zirconia crowns produced through the AM and SM methods demonstrated clinically acceptable marginal fit and ideal hardness exceeding 1200 HV. Some AM groups demonstrated higher hardness and shear bond strength than the SM group. The diverse physical and mechanical properties of various zirconia AM technologies suggest their selective use in specific clinical situations. Certain AM techniques, such as SLA spreading demonstrated comparable or even superior results to those of SM in terms of fit and hardness, indicating their potential as viable alternatives in clinical settings.

## Background

Yttria-stabilized tetragonal zirconia polycrystal (Y-TZP) is a commonly utilized dental restorative material for fixed dental prostheses (FDPs), valued for its mechanical strength, biocompatibility, and aesthetic qualities [1, 2]. Typically, these prostheses are fabricated using computer-aided design and manufacturing (CAD-CAM) technology, whereas the prosthesis is digitally designed (CAD) and then produced through subtractive manufacturing (SM) methods within the CAM process, followed by sintering to achieve full density [3, 4]. The subtractive nature of the SM process presents inherent drawbacks, such as challenges in creating thin internal structures owing to toolpath constraints, excessive material waste, and frequent maintenance requirements resulting from tool wear [5, 6].

To overcome these limitations, additive manufacturing (AM)has emerged as an alternative. AM allows for the precise fabrication of complex internal structures by building materials layer-by-layer, thereby reducing material consumption and offering greater design flexibility compared with SM [6, 7]. The primary AM technologies for zirconia fabrication are digital light processing (DLP) and stereolithography (SLA) [8]. Both methods utilize a light-curable resin within a vat (VAT) for fabrication. DLP controls ultraviolet (UV) light exposure patterns through a digital micromirror device, thereby curing the resin layer-by-layer [9]. DLP utilizes a projector to simultaneously cure an entire layer, thereby offering relatively fast printing speeds. In contrast, SLA selectively cures the cross-sections of the object using a laser beam, allowing for the production of highly intricate patterns in an automated manner [10].

While previous studies on the mechanical properties of zirconia ceramics reported that AM zirconia demonstrates lower mechanical strength compared with SM zirconia [11–13], recent advancements in materials and printing technologies have enhanced the mechanical properties of AM zirconia [14–18]. Moreover, the shear bond strength between zirconia and resin cement is crucial for the long-term success of dental prostheses, ensuring retention and reducing the risk of debinding under functional stresses, such as mastication and thermal cycling. Bond strength can be enhanced by improving the mechanical and chemical interactions at the bonding interface using common surface treatments, such as sandblasting, acid etching, and primer application [19–21]. These characteristics are significant in AM technologies with highly viscous ceramic slurries.

Despite advancements in AM, challenges persist owing to the high viscosity of the slurry, which is a mixture of curable liquids and ceramic particle**s.** Techniques, such as blade spreading and tank polymerization have been introduced to address this issue [22]. Variations in AM technologies and zirconia material compositions can result in variations in hardness, bonding strength, and surface characteristics [23]. Hardness, which represents the resistance of a material to plastic deformation, is crucial to wear resistance and surface durability in dental restorations. Furthermore, hardness testing is non-destructive and highly reproducible, offering practical advantages for comparing the effects of different AM and SM manufacturing processes. Additionally, in the case of definitive FDPs, the fit between the restoration and. Poor marginal fit can result in periodontal disease, underscoring the clinical significance of this factor [24–26].

Therefore, this study aims to explore the various materials and technologies currently utilized in the fabrication of zirconia FDPs using AM compared with those produced through conventional SM processes. The study will evaluate the fit, microhardness, shear bond strength, and surface properties of zirconia FDPs to assess their clinical accuracy, mechanical performance, and physical characteristics. The study hypotheses are as follows: (1) no significant differences will be observed between additively and subtractively manufactured zirconia crowns in these characteristics, and (2) no significant differences will be observed among zirconia crowns produced using different AM methods.

## Materials and methods

### Group classification

In this study, six groups of zirconia crowns were formed, each comprising 12 specimens (*n* = 12), resulting in 72 crowns fabricated using SM (one control group) and AM (five test groups). Two specimens from each group were utilized for surface characterization, whereas the remaining ten were utilized for fit assessment, microhardness, and shear bond strength testing.

The test groups that utilized AM technologies for crown fabrication included four DLP groups and one stereolithography (SLA) group. The DLP groups varied in slurry delivery techniques, shading approaches, and platform movements (Table 1). The DLP circular spreading method involved a downward approach with the platform moving from above to below, whereas all the other groups utilized an upward approach.

**Table 1:** Processing methods and characteristics in this study

Group			Manufacturing method	Machines	Number of specimens
SM		CON	Fabricated using a 5-axis milling machine with SM techniques	M1 (Zirkonzahn SRL, Italy)	10
AM	DLP	DLP spreading	Used a rectangular table and blade for slurry delivery in a uniform color shade	Veltz-Cera90 (Hephzibah Co., Korea)	10
		DLP spreading gradation	Used a rectangular table and blade for slurry delivery with different shades applied layer by layer to create a shade-gradation effect	Veltz-Cera90 (Hephzibah Co., Korea)	10
		DLP vat	Employed a vat-based slurry supply	ZIPRO Dental (Aon Co., Korea)	10
		DLP circular spreading	Used a circular table and blade for slurry delivery	CeraFab System S65 Medical (Lithoz GmbH, Austria)	10
	SLA	SLA spreading	used a rectangular table and blade for slurry delivery	CERAMAKER C900 Flex (3D CERAM SINTO, France)	10
Total					60

#### Design of geometric abutment and crown

**Fig. 1:**
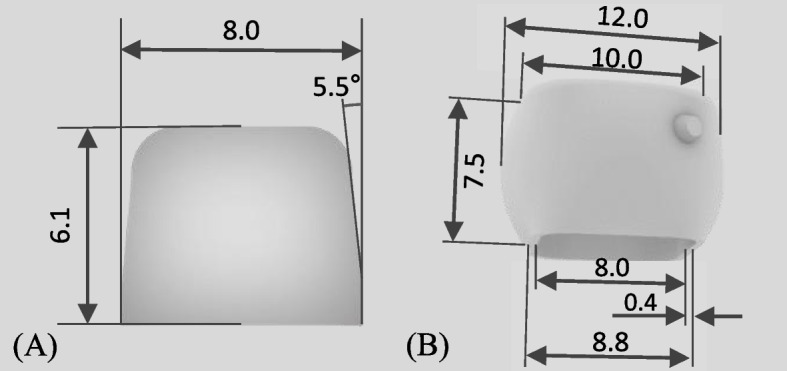
Design dimension (**A**) Abutment, (**B**) External contour of the crown

The abutments and crowns were designed using CAD software (Rhino 7; Robert McNeel & Associates, USA). The abutment dimensions were set at 8 mm length, 8 mm width, and 6 mm height, with a 5.5-degree taper beginning 1 mm above the margin—defined as the internal boundary of the crown-abutment interface [27] (Fig 1). This tailored design was created to assess fit using the replica technique, with a slight gap at the margin and a tapered shape intended to provide a reliable fit for clinical applications. The abutment was fabricated using metal printing with a Co-Cr alloy (Rainbow Metal Printer, Dentium, Seoul, Korea).

In terms of the crown design, the internal dimensions were intentionally made slightly larger than the abutment dimensions. The crown's external shape featured a flat occlusal surface with rounded mesiodistal contours above and below the maximum convexity, mirroring simplified clinical conditions to achieve optimal fit and function.

The cement space was set to 0 mm at the margin and gradually increased to 0.1 mm from 1 mm above the margin. A uniform cement space of 0.1 mm was applied to all other internal surfaces, including the axial and occlusal areas [28]. This design was carefully crafted to replicate real-world clinical scenarios while maintaining the necessary cement space requirements.

#### Crown fabrication

The control group utilized SM methods for crown fabrication, using specimens milled from 4-YTZP zirconia blocks (Prettau; Zirkonzahn SRL, Gais, Italy).

In contrast, the AM group manufactured crowns by importing geometric crown data into the pre-processing software provided by each manufacturer. All crowns were positioned with the occlusal surface facing the bottom edge of the platform, without additional supports, resulting in a 0-degree orientation during the printing process. The layer thickness (z-axis) was set at 50 µm, and data were sliced to generate G-code files. The crowns were produced using DLP and SLA technologies with commercial and in-house pastes. The preset parameters provided by the manufacturers (scraping, hatching, and laser output) were utilized. The uncured paste was removed as per the manufacturer's instructions, followed by debinding and sintering, as recommended. The details of the technologies, paste compositions, and debinding and sintering processes are listed in Table 2.

**Table 2:** Details of the group in this study

Group		SM		AM				
CON		DLP spreading	DLP spreading gradation	DLP vat	DLP circular spreading	SLA spreading		
Materials		Prettau, (Zirkonzahn SRL, Italy)		In-house		ININI-CERA (Aon Co., Korea)	LithaCon 3Y 210 (Lithoz GmbH, Austria)	3DMix ZrO2 (3D CERAM SINTO, France)
Classification by Y2O3 (%)		4Y-TZP		4Y-TZP		Unknown	3Y-TZP	3Y-TZP
Composition (wt%)	ZrO_2_ + HfO_2_		90–94%	85–93%		75–85%	≥ 99.0	Matrix
	Y_2_O_3_		4–8%	6.5–7.5%		15–25%	> 4.5 to ≤ 6.0	Matrix
	Other oxide		≤ 2%	≤ 4.5%		Matrix	≤ 0.5	≤ 0.36
Sintering Protocol		After milling from a solid zirconia block, the restoration was heated at 1500–1600 °C for 4–8 h		After printing, the remaining slurry is removed, washed with ethanol, dried, and sintered at 1450° C for 20 h		Unknown	Preconditioning was done at 120 °C for 134 h, followed by debinding and sintering at up to 1450 °C for 94 h	Debinding was done by gradually heating to 1000 °C and then cooling, followed by sintering at 1450 °C for 20 h

### Fit assessment (Replica technique)

To evaluate the fit between the crown and abutment, the replica technique was employed. The internal surface of the crown was not polished or modified before fit assessment. Fit checker paste type (Fit Checker II, GC Corporation, Tokyo, Japan) was applied to the internal surface of the crown and fitted to the abutment to assess marginal and internal gaps. Each crown was seated for 2 min under a standardized load of 50 N [29], and applied using a calibrated weight by a single operator to ensure consistency. Light-body silicone (Examixfine Injection type, GC Corporation) was utilized to fill the internal space, whereas putty-type silicone (Exafine Putty type, GC Corporation) was used to create a base block for reinforcement.

**Fig. 2:**
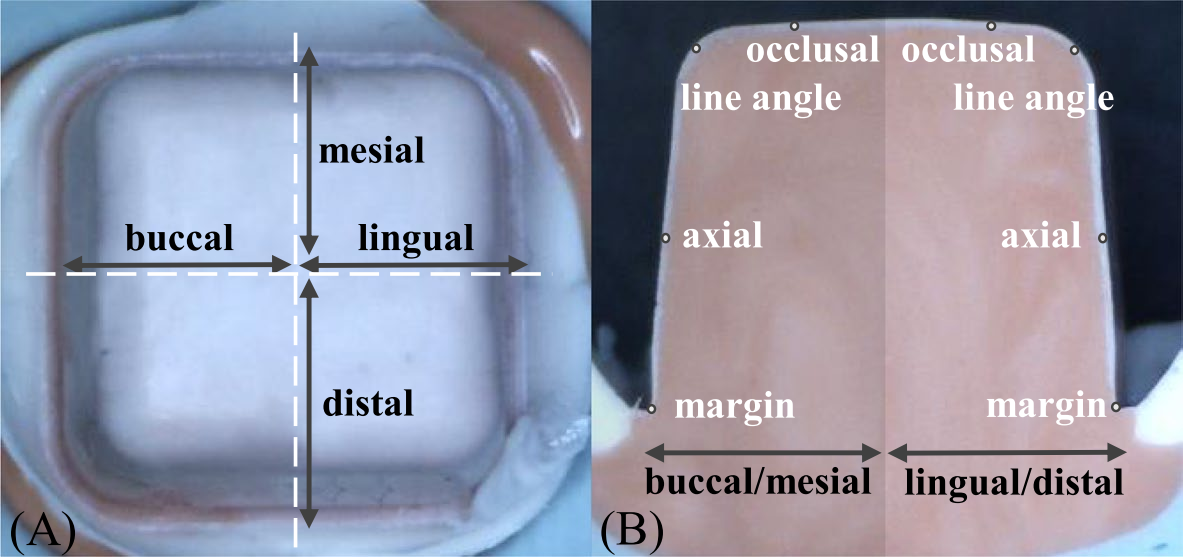
Measurement locations for zirconia crown fit. **A** Sectioning area of the replica specimen, (**B**) Measurement points on the replica specimen

Replica specimens were carefully sectioned both mesio-distally and bucco-lingually to assess gaps at various surfaces including the margin, axial, line angle, and occlusal surfaces (Fig. 2). Measurements were performed at 16 points per crown. Images were captured using a stereomicroscope (SMZ-168-TL, Motic Inc., Wetzlar, Germany) at 30 × magnification. These images were then analyzed using image analysis software (ImageJ ver1.47, NIH, USA).

### Microhardness (Vickers hardness)

Vickers hardness testing was conducted in accordance with international standard ISO 6507–1 using a diamond pyramid indenter with an angle of 136°. The hardness was calculated from the average diagonal length of the indent and the applied load (*P* = kgf) as follows:

$$HV=\frac{1.8544\cdot F}{{d}^{2}}$$

F: Load (kgf).

d: Average diagonal length(mm).

The Vickers microhardness tester (Mitutoyo, Kawasaki, Japan) was utilized with a 1 kgf load applied for 15 s. Each specimen underwent three measurements at different locations on the occlusal surface, specifically at the outer regions, with the average value being utilized as the single hardness value. This rigorous process resulted in 30 hardness tests being performed per group.

### Shear bond strength

Following the hardness tests, the same specimens were subjected to shear bond strength tests. Each crown was embedded in a silicone mold (20 × 20 × 20 mm) filled with a self-curing resin. To ensure optimal bonding, the interface surface was flattened through polishing and subsequently subjected to a surface treatment. Al_2_O_3_ (Renfert GmbH, Germany) was applied at a pressure of 2 bar, followed by etching with a specialized zirconia and ceramic etching solution (smart etching 2 zirconia & ceramic etching solution, S Bio Gold Co., Korea), and finally the application of a zirconia primer (GC Corporation, Tokyo, Japan) as per manufacturer’s instructions.

The center of the occlusal surface, which did not interfere with the Vickers hardness measurements, was designated as the bonding interface. Resin cement (RelyX™ U200, Deutschland, GmbH) was injected into a bonding mold (Ultradent Products, South Jordan, UT, USA) with a diameter of 2.2 mm and height of 2.5 mm. Subsequently, the cement was light-cured (Elipar DeepCure-S, 3 M, St. Paul, MN, USA) before being immersed in distilled water at 37 °C for 24 h.

**Fig. 3:**
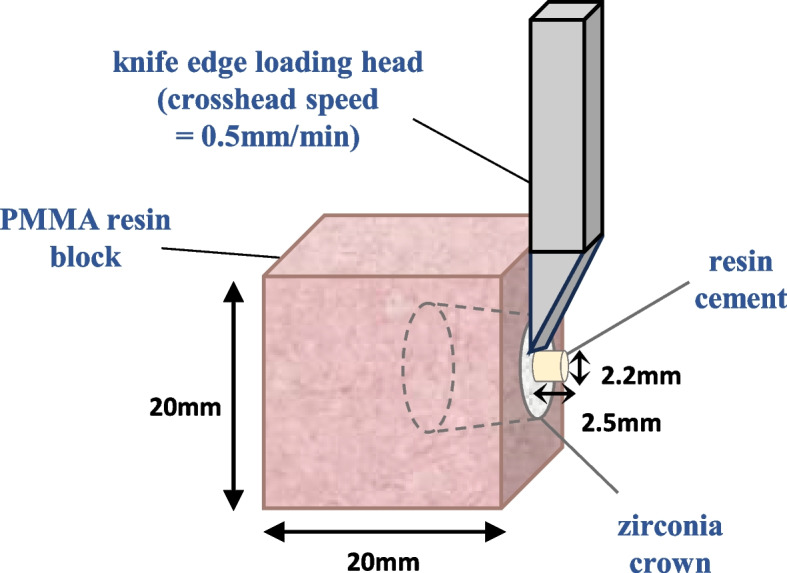
Schematic of the shear bond strength test

A total of 60 specimens were used for the shear bond strength test. The shear bond strength was evaluated using a universal testing machine (TESTONE, Seoul, Korea) equipped with a knife-edge chisel. Tests were conducted at a crosshead speed of 0.5 mm/min until failure occurred (Fig. 3). The shear bond strength was calculated as the maximum force (N) before failure divided by the cross-sectional area of the bonding interface, as follows:

$$MPa=\frac{Load\;at\;failure(N)}{Bonding\;area(3.8\text{mm}^2)}$$

Fracture patterns were observed using a scanning electron microscope (SEM; Apreo2, Thermo Fisher Scientific, Waltham, USA).

### Surface characteristics (SEM analysis)

The surface characteristics of each group were examined using two separate crowns prepared for SEM analysis. One crown was examined immediately after printing with no polishing or sandblasting surface treatments, whereas the other was examined after sandblasting. Specimens were coated with platinum to enhance conductivity and image resolution, and observed using SEM (Apreo2, Thermo Fisher Scientific) at magnifications of 5,000 × and 50,000 ×.

### Statistical analysis

Data were analyzed using statistical software (IBM SPSS v26.0; IBM Corp). Normality tests were conducted for fit, shear bond strength, and microhardness data. The Shapiro–Wilk test indicated that the data were normally distributed for all groups, and Levene’s test validated the homogeneity of variances. Therefore, a one-way analysis of variance (ANOVA) was conducted to assess significant differences among the groups, followed by the Tukey post-hoc test for pairwise comparisons between groups, with a statistical significance (α) of 0.05. ​

To ensure the adequacy of the sample size, a priori power analysis was conducted using G*Power 3.1.9.4. Based on a medium effect size (f = 0.40), α = 0.05, and power = 0.80, the required estimated sample size for a one-way ANOVA with six groups was 66. Although the study included 60 specimens (10 per group), considering the anticipated effect size and practical constraints, the selected sample size was deemed appropriate and yielded statistically significant results.

## Results

### Fit assessment (Replica technique)

The mean and standard deviation of the marginal and internal fit measured at each point on replicas obtained from the SM and AM groups are listed in Table 3. The analysis of marginal fit revealed that the SLA spreading technique showed the smallest gap across all evaluated regions, indicating the highest level of adaptation. Conversely, the DLP Vat and DLP circular spreading groups showed significantly larger gaps compared with the control (CON) group and other AM groups (*P* < 0.001).

**Table 3:** Marginal and internal gap of each group (µm)

	CON	DLP spreading	DLP spreading gradation	DLP vat	DLP circular spreading	SLA spreading	**F**	**df**	***p***
Margin	48.45 (28.71)Aa	53.57 (29.23)Aa	61.98 (32.56)Aa	67.90 (27.86)Ab	81.95 (34.36)Bb	42.83 (34.36)Aa	9.75	5	< 0.001
Axial	121.88 (34.45)Bb	132.23 (51.22)Bb	156.65 (65.46)Bc	112.80 (32.00)Bb	133.60 (38.65)Db	82.25 (19.62)Ba	12.47	5	< 0.001
Line angle	178.23 (38.52)Cb	193.13 (53.55)Cb	193.05 (66.84)Cb	237 (69.74)Cc	123.85 (30.25)Ca	88.13 (22.10)Ba	46.34	5	< 0.001
Occlusal	198.48 (19.68)Db	224.70 (28.16)Db	189.95 (40.55)Cb	384.43 (96.47)Dc	58.13 (18.19)Aa	87.83 (23.51)Ba	246.77	5	< 0.001
**F**	186.12	127.38	52.33	202.02	51.52	45.63			
**df**	3	3	3	3	3	3			
***P***	< 0.001	< 0.001	< 0.001	< 0.001	< 0.001	< 0.001			

*df* degrees of freedom, *P* *p*-value

The numbers in parentheses are the standard deviations

The means within the same columns with different uppercase in each parameter are significantly different (*p* < 0.05), and the means within the same rows with different lowercase in each parameter are significantly different (*p* < 0.05)

### Microhardness (Vickers hardness)

The Vickers hardness measurements of zirconia specimens indicated the following order of hardness: DLP spreading, DLP spreading gradation, DLP circular spreading, CON, DLP Vat, and SLA spreading. Statistically significant differences were observed among the groups (*P* < 0.001) (Table 4).

**Table 4:** The results of Vickers hardness test (HV)

CON	DLP spreading	DLP spreading gradation	DLP vat	DLP circular spreading	SLA spreading	**F**	**df**	***p***
1473.87 (61.18)ab	1532.53 (35.95)c	1501.83 (57.90)bc	1462.21 (39.65)ab	1510.37 (22.69)bc	1441.94 (27.95)a	6.030	5	< 0.001

*df* degrees of freedom, *P* *p*-value

The means within the same rows with different lowercase letters in each parameter are significantly different (p 0.05)

### Shear bond strength

**Fig. 4:**
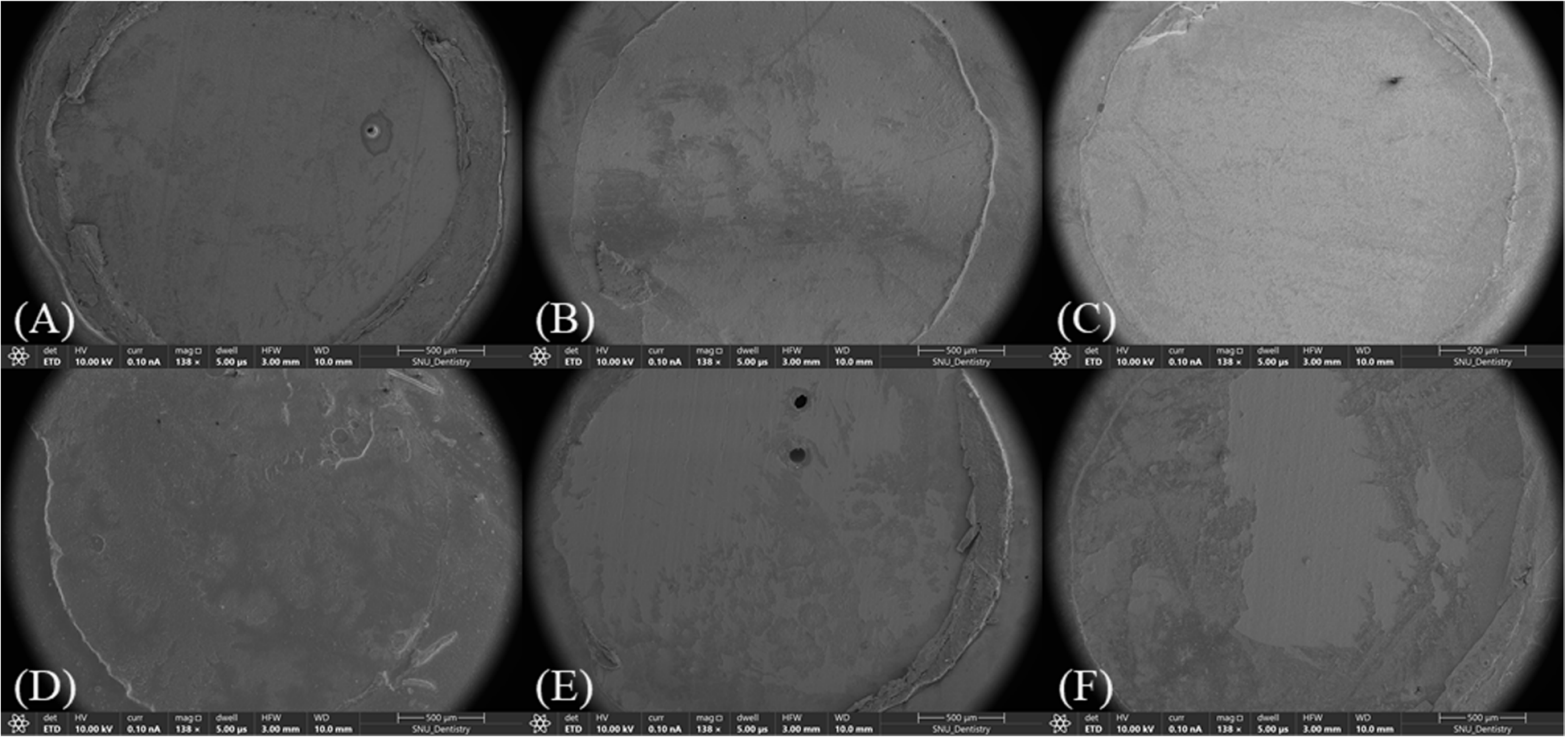
Scanning electron microscopy images of fractured zirconia specimens following the shear bond strength test. Adhesive failure was observed in all specimens across the groups. (magnification: 138 ×). (**A**) CON, (**B**) DLP spreading, (**C**) DLP spreading gradation, (**D**) DLP vat, (**E**) DLP circular spreading, (**F**) SLA spreading

The average and standard deviations of the shear bond strength of the resin cement applied to the zirconia surfaces are listed in Table 5. The DLP spreading gradation group demonstrated the highest shear bond strength (8.97 ± 5.73 MPa), whereas the DLP spreading group demonstrated the lowest shear bond strength (6.97 ± 5.73 MPa). However, no significant differences were observed between the groups. Examination of the fracture surfaces using SEM revealed adhesive failure patterns across all groups (Fig. 4).

**Table 5:** The overall results of the shear bond strength values (MPa)

CON	DLP spreading	DLP spreading gradation	DLP vat	DLP circular spreading	SLA spreading	**F**	**df**	***p***
7.97 (3.24)	6.97 (3.47)	8.97 (5.73)	7.93 (4.55)	7.79 (4.17)	7.59 (4.21)	0.228	5	0.949

*df* degrees of freedom, *P* *p*-value

### Surface characteristics (SEM analysis)

**Fig. 5:**
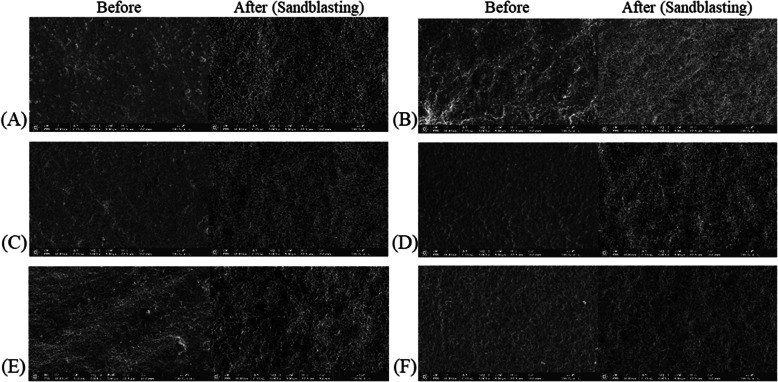
Scanning electron microscopy observation and surface analysis (at 5,000X). (**A**) CON, (**B**) DLP spreading, (**C**) DLP spreading gradation, (**D**) DLP vat, (**E**) DLP circular spreading, (**F**) SLA spreading

**Fig. 6:**
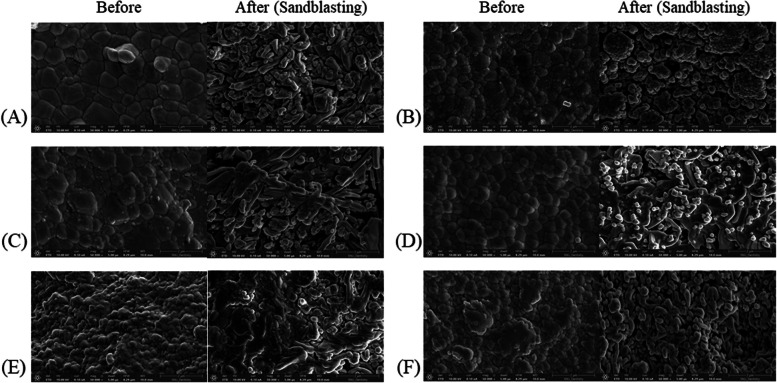
Scanning electron microscopy observation and surface analysis (at 50,000X). (**A**) CON, (**B**) DLP spreading, (**C**) DLP spreading gradation, (**D**) DLP vat, (**E**) DLP circular spreading, (**F**) SLA spreading

The SEM images of the specimens from each group are shown in Fig. 5 (5,000 ×) and Fig. 6 (50,000 ×), demonstrating the surface morphology before and after sandblasting. SM resulted in uniform surfaces, whereas the AM groups demonstrated irregularities. At a higher magnification (Fig. 6), the zirconia particles were clustered together. Following sandblasting, the surfaces demonstrated increased roughness, with deep and wide grooves and relatively small scattered particles distributed across the surface, particularly in the AM groups.

## Discussion

This study analyzed the accuracy, mechanical properties, and surface characteristics of zirconia crowns manufactured using SM and AM techniques. The findings revealed variations in fit, surface characteristics, and microhardness among the manufacturing techniques, resulting in the partial rejection of the first and second hypotheses.

The replica technique was employed to measure the gaps between the crowns and abutments to evaluate the fit of zirconia crowns manufactured using the two techniques. A larger marginal gap can facilitate bacterial invasion and plaque accumulation, potentially leading to secondary caries or periodontal disease. The marginal fits recorded in this study ranged from 42.83 to 81.95 μm, falling within the clinically acceptable range proposed by previous studies. McLean et al. [25] established a clinical marginal fit of 120 μm as the benchmark. The marginal fits in this study were lower than those proposed in previous studies, indicating that they were within an acceptable clinical range. Notably, significant differences in marginal fit were observed between the SM (CON; 48.45 ± 28.71 μm) and AM groups (DLP Vat; 67.90 ± 27.86 μm, DLP circular spreading; 81.95 ± 34.36 μm). These variances are likely attributed to factors in the zirconia AM process that impact the marginal fit, such as printing speed, material viscosity, and shrinkage during curing [30, 31].

Significant differences were observed within the AM groups, particularly between the DLP Vat and DLP circular spreading groups compared with other AM groups (DLP spreading; 53.57 ± 29.23 μm, DLP spreading gradation; 61.98 ± 32.56 μm, SLA spreading; 42.83 ± 34.36 μm). These findings suggest that discrepancies in fit and accuracy can arise even within the same DLP technology owing to variations in printing parameters and post-processing sintering conditions. Various factors, such as the type of projector employed to reduce pixel size, adjustments in pixel resolution, interlayer spacing, and control of sintering profiles, influence the final fit of the printed prostheses [32, 33]. Therefore, optimizing these variables within DLP systems could potentially enhance the clinical performance of AM restorations. Despite being fabricated using the same equipment, the DLP spreading and DLP spreading gradation groups demonstrated differing marginal fit results. SLA spreading group demonstrated the smallest discrepancies across all sites, showing the best fit. This is consistent with previous studies reporting that the SLA method offers superior precision compared with DLP-based technologies [34] and the SM method [35]. Our findings support the clinical viability of SLA technology, and superior marginal fit plays an important role in the clinical longevity and functional stability of dental prostheses [19, 31, 36, 37].

Despite utilizing identical zirconia powders and solid content ratios, the group employing a gradient slurry with multiple colorants demonstrated greater variability in fit [38, 39]. This discrepancy may be attributed to changes in the particle characteristics and slurry rheology introduced by the color additives, as well as increased complexity in the layering process involving multiple material sources. In particular, variations in light absorption owing to the presence of different colorants could result in uneven photopolymerization, leading to discrepancies that accumulate throughout the printing process and ultimately impact the overall accuracy of the restoration the overall accuracy of the restoration.

Except for the DLP circular spreading group, all other groups demonstrated a tendency for an increase in the internal gap from the axial wall toward the line angle and occlusal areas. This trend can be attributed to the unique manufacturing characteristics of each technology. In the case of SM, this pattern may be attributed to tool compensation applied to inaccessible regions during zirconia block milling. In such instances, areas that cannot be properly milled are over-trimmed to compensate for the tool's limitations [40]. AM constructs structures in the Z-direction and the observed discrepancies can be attributed to cumulative errors during the layering process [41]. These errors often manifest as mismatches or stair-step artifacts at the interface between layers, particularly in regions with abrupt geometric transitions, such as the line angle. Furthermore, variations in light intensity, layer thickness, polymerization time, and resin viscosity can influence the extent and precision of curing, potentially resulting in either over- or under-polymerization, which in turn impacts internal fit [42, 43]. Moreover, the simplified geometry of the specimens utilized in this study may have influenced the results. The specimens had a cross-sectional shape resembling a square with relatively sharp edges rather than rounded contours, potentially causing the fit checker material to remain in the occlusal area during the replica fabrication process. Therefore, further studies utilizing specimens that better reflect clinical conditions are necessary.

Hardness is an indicator of a material's ability to resist plastic deformation when subjected to an external force [37, 44], and it is closely related to wear resistance [45]. In the context of zirconia prostheses, lower hardness levels can potentially compromise their stability during clinical use [46, 47]. Vickers microhardness testing revealed that the SM group demonstrated a hardness of 1473.8 HV, whereas that of the AM groups ranged from 1441.94 to 1532.53 HV, with some groups demonstrating comparable or even higher hardness levels compared with the SM group. The ideal hardness for zirconia prosthetics is above 1,200 HV (ISO 6872:2015), and all groups in this study exceeded this value. Our finding aligns with those of recent studies [48, 49], suggesting that the AM technology offers hardness performance that is on par with or superior to conventional milling methods. However, in this study, surface hardness measurements were only obtained for the specimens printed at a 0-degree orientation. The mechanical properties of AM outcomes can be influenced by printing orientation. Therefore, caution must be exercised when extrapolating these results, as variations in printing angles could influence the mechanical properties and surface characteristics [50].

The shear bond strength between the zirconia and resin cement was evaluated using a method that accounted for specimen shape [20, 21]. Bonding molds were utilized to ensure consistent application of the resin cement. Examination of the fracture surface revealed adhesive failure across all groups. Despite employing surface treatments, such as sandblasting and priming to enhance mechanical and chemical bonding, the inherent strength of zirconia likely contributed to interface separation. Previous studies also observed adhesive failure with zirconia owing to its low surface energy and dense surface structure [19–21]. The shear bond strengths of the resin cement were 7.97 MPa for the milling specimens and 6.97–8.97 MPa for AM specimens, with no significant differences observed between the two manufacturing techniques. Notably, this study was conducted in vitro, and the use of a simplified design for resin cement applications may present challenges in accurately assessing bonding capabilities based solely on shear bond strength. Therefore, future clinical studies should aim to measure bonding strength and examine fracture surfaces to obtain more realistic results.

The SEM images provided insights into the varying sizes of agglomerates present in each group. These agglomerations can be attributed to the high surface energy of the zirconia nanoparticles in the slurry, which leads to aggregation. Li et al. [51] reported that the high surface energy of nanoparticles promotes interactions between particles, resulting in agglomerate formation. Notably, finer powders with higher surface area and energy may influence particle size and distribution [52]. Uniform blocks with minimal height variations were observed in the SM group. The zirconia blocks utilized in the milling were compressed into dense, uniform structures, resulting in a consistent and compact surface. This uniformity was maintained during machining, resulting in flat surfaces with limited microstructural variation. In contrast, the AM groups demonstrated more contoured surfaces and irregular particle distribution, likely resulting from the high viscosity of the zirconia slurry and the layer-by-layer polymerization process inherent to AM.

Following sandblasting, all the groups displayed increased surface roughness; however, the extent of change varied depending on the initial surface conditions. At higher magnifications, the milled specimens maintained structural stability and demonstrated relatively moderate alterations, whereas the DLP-based groups (spreading, vat, and circular) demonstrated more pronounced microstructural changes, including deeper grooves and greater surface disruption. Furthermore, these groups demonstrated less densely packed structures prior to sandblasting. The SLA specimens showed smoother and more uniform surfaces with less pronounced agglomeration and finer surface textures, which remained relatively well-preserved even after sandblasting. However, the observed differences at higher magnifications may be attributed to intrinsic material features, such as the degree of compaction and sintering behavior [53], rather than solely to the printing technology itself. These variations in the post-processed surface features could have implications for micromechanical retention, adhesive behavior, and long-term prosthetic performance.

This study had several limitations. First, the surface analysis was qualitative rather than quantitative, limiting the ability to compare the surface characteristics numerically. Second, the study focused on single-unit prostheses and did not assess multi-unit restorations or simulate complex clinical conditions, potentially restricting the generalizability of the findings. The current research on AM zirconia sets the stage for future advancements in materials and technologies. The testing of various AM techniques and comparison of their results with those of conventional SM methods is crucial. Future research endeavors should focus on testing more zirconia printing technologies, incorporating additional variables, such as printing orientation and thermal treatment methods, and exploring the effects of physical variables, such as sandblasting on the mechanical properties.

## Conclusion

Both the SM and AM techniques produce zirconia crowns with marginal fits within the clinical acceptance range. Some AM techniques have even demonstrated zirconia hardness levels that are comparable with or surpass that of traditional SM, with all techniques achieving a hardness of over 1,200 VHN. The shear bond strength results were consistent across both the SM and AM methods. SEM images revealed distinct types of agglomerates among the groups. The SM group demonstrated uniform surfaces, whereas the AM groups demonstrated contoured shapes. These findings suggest that certain AM techniques, such as SLA spreading, may offer clinically viable alternatives to conventional SM methods, demonstrating comparable or even superior performance in specific aspects.

## Data Availability

Data is provided within the manuscript or supplementary information files.

## Abbreviations

Y-TZP: Yttria-stabilized tetragonal zirconia polycrystal
FDPs: Fixed dental prostheses
CAD-CAM: Computer-aided design and manufacturing
CAD: Computer-aided design
SM: Subtractive manufacturing
AM: Additive manufacturing
DLP: Digital light processing
SLA: Stereolithography
VAT: At-based system
UV: Ultraviolet
HV: Vickers hardness
ISO: International Organization for Standardization
